# srGAP1 mediates the migration inhibition effect of Slit2-Robo1 in colorectal cancer

**DOI:** 10.1186/s13046-016-0469-x

**Published:** 2016-12-07

**Authors:** Yuyang Feng, Lei Feng, Di Yu, Jian Zou, Zhaohui Huang

**Affiliations:** 1 Wuxi Oncology Institute, Affiliated Hospital of Jiangnan University, 200 Hui He Road, Wuxi, Jiangsu 214062 China; 2 Wuxi Medical School, Jiangnan University, Wuxi, Jiangsu 214122 China; 3 Department of Clinical Laboratory Science, Wuxi People’s Hospital of Nanjing Medical University, 299 Qingyang Road, Wuxi, Jiangsu 214023 China

**Keywords:** Slit2, Slit-Robo Rho GTPase activating protein 1 (srGAP1), Cell migration, Colorectal cancer

## Abstract

**Background:**

The neuronal guidance molecule Slit2 plays suppressive role in tumorigenesis and progression. We previously showed that Slit2-Robo1 inhibit cell migration in colorectal cancer (CRC). However, little is known about its downstream effectors in CRC. This study tries to identify whether the Slit-Robo Rho GTPase activating protein 1 (srGAP1) could mediate the inhibitory effect of Slit2-Robo1 on CRC cell migration.

**Methods:**

The protein expression of srGAP1 in clinical CRC tissues was tested by immunohistochemistry staining. Conditioned medium was prepared from HEK293 cells stably expressing Slit2-myc, Robo1-HA or RoboN (a soluble extracellular domain of Robo1). Immunoprecipitation (IP) was applied to check the interaction between Robo1 and srGAP1, and immunofluorescence (IF) was used to observe the subcellular localization of Robo1 and srGAP1. Small GTPase pull-down assay was used to determine the activity of Cdc42. A modified wound healing assay was performed to detect cell migration.

**Results:**

The protein expression of srGAP1 was remarkably decreased in 47.5% of CRC tissues compared with adjacent noncancerous tissues, and the decreased srGAP1 expression was associated with lymphatic invasion, poor tumor differentiation, high TNM stage, and poor survival (*P* < 0.05). IP and IF assays revealed that srGAP1 was a Robo1-interacting protein and exhibited similar dynamic subcellular distribution after Slit2 treatment in CRC cells. Small GTPase pull-down assay and migration assay indicated that Slit2-Robo1 signaling inhibited Cdc42 activity and CRC cell motility through srGAP1.

**Conclusion:**

Downregulation of srGAP1 in CRC was associated with tumor progression and poor prognosis. srGAP1 is an important downstream molecule of Slit2 signalling in CRC, and mediates the anti-migration function of Slit2 by inhibiting Cdc42.

## Background

Colorectal cancer (CRC) is one of the most common cancer worldwide [1]. The incidence of CRC persistently increases in China and other developing countries, and CRC remains as an important health concern worldwide [2]. High motility ability of CRC cells is a key factor to prompt tumor metastasis, which is one of the key reasons resulting in therapy failure and cancer-related death.

Tumor progression requires increased cell motility, which is accompanied by increased actin polymerization and enhanced activity of proteins that optimize its turnover. Previous studies have showed that Slit-Robo signaling affects cell motility by controlling the activity of several proteins involved in reorganizing the actin cytoskeleton, including the Rho GTPases family (Rac, Cdc42, and RhoA) [3].

Slits are secreted extracellular matrix proteins expressed in many types of cells and tissues. As the key neuronal guidance molecules, Slits were originally identified to regulate axonal guidance and neuronal migration in the central nervous system by binding to Roundabout receptor family (Robo1-Robo4) [4, 5]. In addition to their functions in nervous system, recent data revealed that Slit-Robo pathway also plays key roles in human tumorigenesis and progression [3].

Slit2 is the most common studied member of Slit family and exerts its activity mainly by binding to Robo1. Slit2 is frequently downregulated in many types of cancers and exhibits tumor suppressive functions, especially inhibition of tumor cell migration. Although Zhou et al. [6] reported that Slit2 could prompt CRC tumorigenesis and metastasis, our recent work showed that Slit2 is frequently downregulated in clinical CRC tissues and inhibits CRC cell migration in a Robo-dependent manner [7]. Dallol et al. [8] and Chen et al.[9] also reported the tumor suppressive role of Slit2 in CRC. However, little was known about the detailed regulation mechanism of Slit2-Robo1, especially its downstream targets in CRC.

The Slit-Robo Rho GTPase activating protein 1 (srGAP1) is a key GTPase activating protein (GAP) downstream of Slit-Robo pathway, and has been shown to inhibit neuronal migration [10] and glioma cell invasion [11] by reducing the activation of Cdc42. Whether srGAP1-Cdc42 axis is a key downstream functional target of Slit2 signaling in CRC remains to be elucidated and little was known about the expression and clinical significance of srGAP1 in human cancer.

In this study, we identify that srGAP1 is a key Robo-interacting protein and mediates the migration inhibitory function of Slit2 by inhibiting Cdc42 activity in CRC. Clinical sample analyses revealed that srGAP1 was significantly downregulated in CRC tissues, which was associated with tumor progression and poor patient survival.

## Methods

### Tumor tissues and cell lines

A total of 156 human primary CRC tissues and their adjacent noncancerous tissues (NCT) were collected with informed consent at Affiliated Hospital of Jiangnan University, and the detailed clinical information of these patients was included in Table 1. This project was approved by the Clinical Research Ethics Committee of the Affiliated Hospital of Jiangnan University.

**Table 1:** srGAP1 protein expression in CRC tissues

Characteristics	Number	srGAP1 score		*p* value
		0 or 1	2 or 3	
Ages (years)				
≥ 65	76	44	32	0.602
< 65	80	43	37	
Gender				
Male	70	39	31	0.990
Female	86	48	38	
Tumor size (cm)				0.733
< 5	79	43	36	
≥ 5	77	44	33	
Differentiation				**0.024**
Well	14	3	11	
Moderately	110	64	46	
Poorly	32	20	12	
Nodal status				
Positive	78	36	42	**0.016**
Negative	78	51	27	
TNM stage				
I + II	75	34	41	**0.012**
III + IV	81	53	28	

Human CRC cell lines (HCT8, HCT116, LoVo, Caco2, DLD1, and HT29) and HEK293 cell line were purchased from American Type Culture Collection (ATCC). All of the culture media (Hyclone, USA) were supplemented with 10% fetal bovine serum (FBS) (Clark, Australia). These cells were incubated under the conditions recommended by ATCC.

### Antibodies and plasmids

Mouse anti-β-actin (A1978), anti-β-tubulin (T8328) and anti-Flag (F1804) from Sigma-Aldrich (USA); mouse anti-HA (16B12, MMS-101R), anti-myc (9E10, MMS-101R) and rabbit anti-GFP (NB600-308) from Covance Laboratories Ltd (UK); anti-Cdc42 from BD Transduction Laboratories (No.610929, USA); anti-Robo1 from Abcam (ab7279, USA), anti-srGAP1 from Proteintech (13252-1-AP, USA). The plasmid constructs of Robo1-HA, Robo1-myc, Robo1-RFP, RoboN (a soluble extracellular domain of Robo1), srGAP1-Flag, srGAP1-GFP, srGAP1ΔGAP (Δ320–570), GFP-USP33, CACdc42 (constitutively active), DNCdc42 (dominant negative), and GST-PAK1 were generated as described [5, 10, 12]. Plasmid transfection was performed using Lipofectamine 2000 (Invitrogen, USA).

### Immunohistochemistry (IHC) staining

Tissue arrays were constructed using 156 paired CRC tissues and NCTs. The protein expression of srGAP1 was measured by IHC method that was performed on 4 μm paraffin-embedded CRC tissue sections. In brief, the slides were hatched with anti-srGAP1 antibody at a dilution of 1:100 overnight at 4 °C, followed by 1 h incubation of secondary antibody. The subsequent steps were performed using GTvision^TM^ III Detection System/Mo&Rb (DAKO, USA). The protein expression of srGAP1 was scored 0, and 1, 2, and 3 according to the percentage and staining intensity of positive colorectal epithelium cells.

### Conditioned medium (CM)

HEK293 cells stably expressing Slit2-myc, Robo1-HA or RoboN were established as previously described [5]. To generate CM, these HEK293 stable cells were plated in 10 cm^2^ dishes and grown to approximately 80% confluence in normal growth medium, and were then incubated in 8 mL of DMEM medium supplemented with 5% FBS for 48 h. The supernatant was collected, centrifuged and then stored in −80 °C until use. In some experiments, CM was concentrated using Amicon Ultra centrifugal filtration devices (Millipore, USA), and working concentrations of Slit2 in CM was estimated to be 100 ~ 250 ng/mL according to the silver staining of serial dilutions of CM following SDS-PAGE fractionation.

### RNA extraction and RT-PCR

Total RNA was extracted using the RNAiso Plus (Takara, Japan) according to the manufacturer’s instructions. The concentrations of RNA were measured by NanoDrop ND-2000 instrument (NanoDrop, USA) and complementary DNA (cDNA) was synthesized using the HiFiScript cDNA Kit (CWBIO, China). RT-PCR was used to detect gene expression in CRC cell lines, with β-actin as an internal control. The primers used were described in our previous study [7].

### Western blot and immunoprecipitation (IP)

Western blot was performed as previously described [7], and the dilution ratios for different primary antibodies were 1:3000 (anti-β-actin, anti-β-tubulin, anti-Flag, anti-HA, and anti-myc), 1:2000(anti-GFP), 1:1000(anti-Cdc42, anti-Robo1 and anti-srGAP1). For IP assay, plasmid constructs of Robo1-HA, srGAP1-GFP, srGAP1-Flag, USP33-GFP or control vector were transfected into CRC cells. Forty-eight hours after transfection, cells were lysed using IP lysis buffer (1% Triton-100, 10% Glycerol, and 1 mM EDTA in TBS buffer), and IP was performed using anti-HA or anti-Flag as described previously [5].

### Immunofluorescence (IF)

CRC cells were cotransfected with plasmids of Robo1-RFP, srGAP1-GFP or control. Forty-eighty hours after transfection, cells were treated with mock or Slit2 CM for 10 min and were then fixed with 4% paraformaldehyde at room temperature for 30 min. The images were obtained with an inverted fluorescence microscope (Olympus, Japan).

### Small GTPase pull-down assay

GST-PAK1 fusion protein was expressed in E. coli (BL21 strain), and purified by glutathione-sepharose affinity chromatography. Purified GST-PAK1 protein was incubated with GST-sepharose beads and used for GST pull-down assay to detect the GTP-loading of Cdc42 (GTP-Cdc42).

To study the potential regulation of Slit2 on Cdc42 activity in CRC, different plasmids were transfected into CRC cells. Forty-eighty hours after transfection, cells were lysed in ice-cold IP lysis buffer and were subjected to GST pull-down assay. Briefly, total cell lysates were incubated with GST-sepharose beads loaded with GST-PAK1 protein at 4 °C for 2 h. The beads were washed three times with lysis buffer. Bounded proteins were eluted by incubation in sample buffer at 95 °C for 10 min and separated by 15% polyacrylamide gel electrophoresis. Bounded Cdc42 was analyzed by Western blot using an anti-Cdc42 antibody.

### Cell migration assay

A modified wound healing assay was performed to detect cell migration as we described previously [7]. Briefly, photo-etched grid coverslips (Bellco Biotechnology, USA) were coated with 10 μg/mL poly-L-lysine, and cells were then seeded on the coated coverslips and grown to confluence in the 3.5 cm dish. A wound scratch was made by scraping a micropipette tip across the cell monolayer. After removing the dislodged cells by gentle washing with culture media, cells were treated with CM for 10 h. At 0 h and 10 h after wound formation, phase-contrast images of the wound-healing process were photographed under an inverted microscope. Ten representative images were taken at the corresponding positions marked by letters on the etched grid coverslips, and the directional cell migration was measured as the forward distance that individual cells migrated from the wound edge line at the zero time point.

### Statistical analysis

The results are presented as the mean values ± SEM. The data were subjected to χ^2^ test, Student’s t-test, the Mann–Whitney U test or the Spearman Rank correlation test. Kaplan-Meier survival analyses and the log-rank test were carried out to determine differences in overall survival (OS). The Cox proportional hazards model was used to estimate hazard ratios (HRs) and their 95% confidence intervals (CIs), representing the overall relative risk of death. A *P* value of less than 0.05 was considered statistically significant. The Graphpad prism 5.0 software (GraphPad Software, USA) and SPSS 20.0 package (IBM, USA) were used for the statistical analyses and scientific graphing, respectively.

## Results

### srGAP1 is downregulated in CRC and is associated with poor survival

**Fig. 1:**
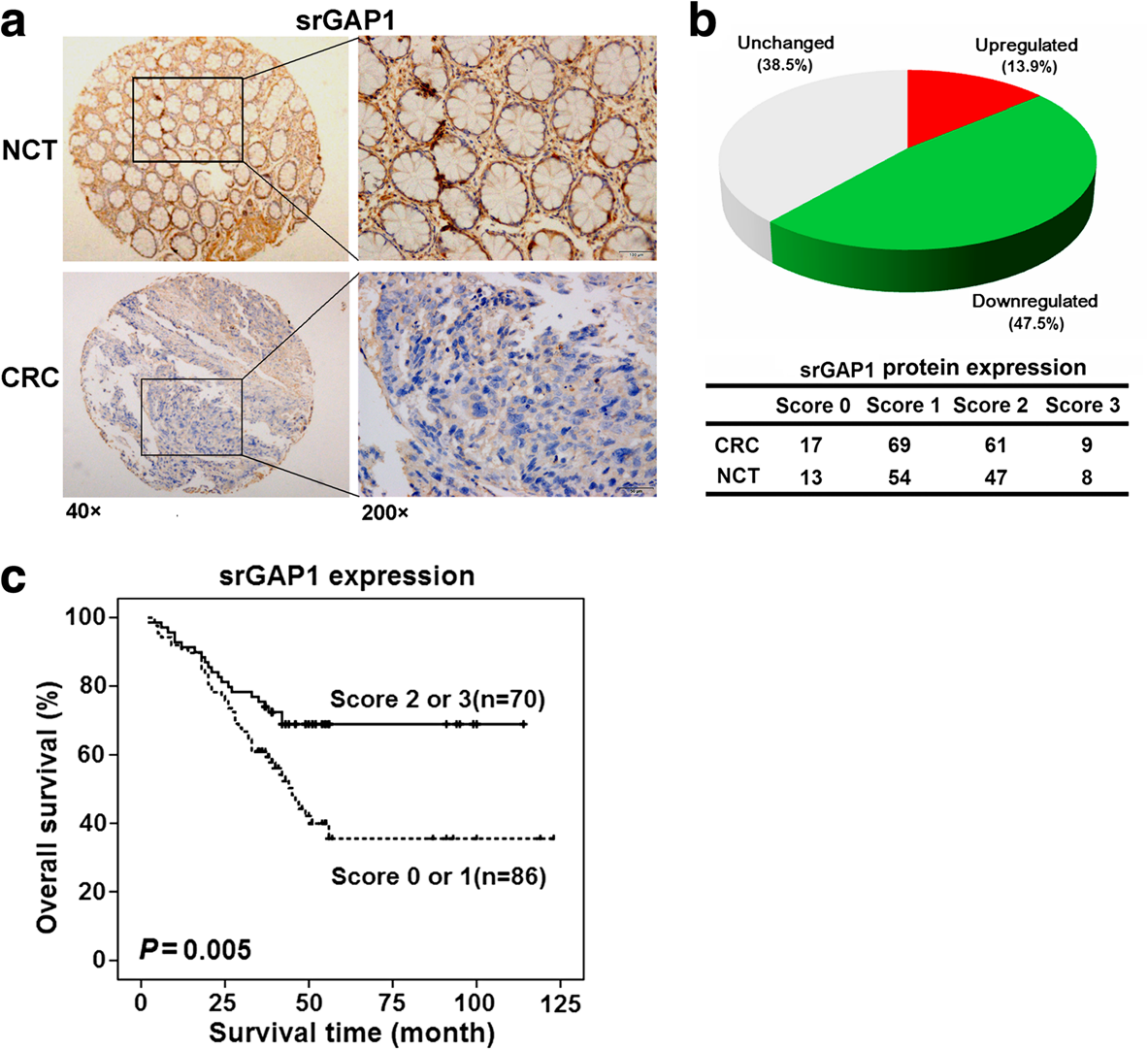
srGAP1 is downregulated in CRC tissues and correlates with poor survival. **a** Representative images of IHC staining of srGAP1 in CRC tissues and adjacent NCTs. **b** The protein expression of srGAP1 was frequently decreased in 47.5% of CRC tissues compared with paired NCTs. The staining results of srGAP1 protein were available in 156 CRC and 122 NCT tissues. **c** Overall survival analysis based on the protein expression levels of srGAP1 in CRC. CRC patients with low srGAP1 levels (score 0 or 1) had a significantly poorer OS than patients with high srGAP1 levels (score 2 or 3)

Little was known about the expression and clinical significance of srGAP1 in human cancers, including CRC, so we measured the protein levels of srGAP1 in 156 paired CRC and NCT tissues by IHC staining. The srGAP1 expression results were not available in 34 NCT samples due to technical difficulty in the tissue microarray construction and IHC staining. As shown in Fig. 1a, srGAP1 was expressed mainly in cytoplasm and was significantly downregulated in 47.5% (58 of 122) of CRCs compared with NCTs (Fig. 1b). To evaluate the clinical significance of srGAP1 in CRC, the potential relationships between srGAP1 expression and patients’ clinicophathological characteristics were analyzed (Table 1). Noticeably, srGAP1 expression in CRCs was significantly correlated with tumor differentiation (Spearman *r* = −0.162, *P* = 0.044), lymphatic invasion (Spearman *r* = −0.194, *P* = 0.015) and TNM stage (Spearman *r* = −0.202, *P* = 0.011), suggesting that srGAP1 expression was associated with tumor progression.

Survival analysis showed that patients with low srGAP1 expression (score 0 or 1) had a significantly poorer OS than patients with high srGAP1 expression (score 2 or 3) (χ^2^ = 7.815, *P* = 0.005, Fig. 1c). Univariate analysis on these 156 CRCs showed that relative low srGAP1 expression, positive nodal status, poor differentiation and higher TNM stage were correlated with decreased survival time (Table 2). The four parameters were then subjected to a Cox multivariate analysis. After adjusting for nodal status, tumor differentiation and TNM stage, multivariate analyses revealed that patients with high srGAP1 expression show a trend of prolonged survival time as compared to patients with low srGAP1 expression (HR = 0.613; 95% CI, 0.360–1.041, *P* = 0.070) (Table 2). Collectively, these data suggest the tumor suppressive role of srGAP1 in CRC.

**Table 2:** Univariate and multivariate analyses of OS for the 156 CRC patients

	Univariate analysis			Multivariate analysis		
	*P*	HR	95% CI	*P*	HR	95% CI
Ages (<64 / ≥64)	0.071	1.567	0.962–2.551			
Gender (male/female)	0.404	0.815	0.505–1.317			
Tumor size (<5 cm / ≥5 cm)	0.817	0.945	0.585–1.527			
Nodal status (negative/positive)	0.000	2.823	1.689–4.719	0.050	0.299	0.089–1.002
Differentiation (well or moderately/poorly)	0.000	3.358	2.004–5.625	0.002	2.442	1.393–4.281
Tumor stage (I + II/ III + IV)	0.000	3.342	1.956–5.711	0.001	7.529	2.167–26.156
srGAP1 expression (low/ high)	0.006	0.487	0.290–0.817	0.070	0.613	0.360–1.041

*HR* hazard ration, 95%, *CI* 95% confidence interval

### Slit2, Robo1 and srGAP1 expression in CRC cell lines

**Fig. 2:**
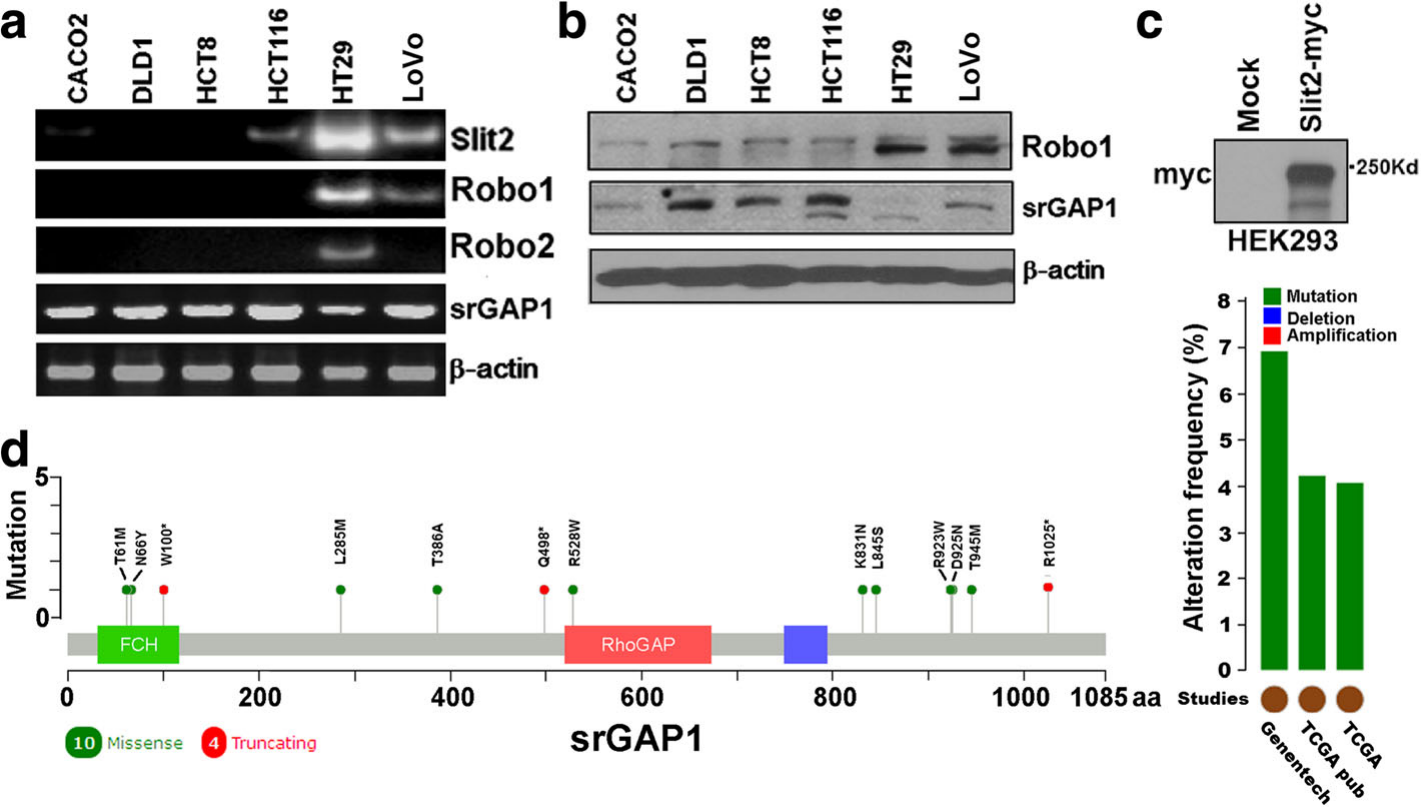
Slit2 inhibits CRC cell migration in a Robo dependent manner. **a** The mRNA expression of components in the Slit/Robo signaling in CRC cell lines. **b** The protein expressions of Robo1 and srGAP1 in CRC cell lines were detected by western blot, with beta-actin as a loading control. **c** Detection of Slit2-myc in slit2 conditional medium (CM). **d** Mutation at srGAP1 locus was analyzed based on data obtained from the Stand Up to Cancer cBio portal (http://cbio.mskcc.org/su2c-portal/)

The mRNA levels of Slit2, Robo1 and srGAP1 were detected in CRC cell lines using RT-PCR. The results revealed that both Slit2 and Robo1 were expressed in HT29 and LoVo cells, whereas srGAP1 mRNA expressed in all these six CRC cell lines with lowest expression in HT29 cells (Fig. 2a). Western blot results confirmed the Robo1 expression in HT29 and LoVo cells, whereas no detectable srGAP1 protein was observed in HT29 cells (Fig. 2b). Based on these results, we focused on LoVo cell line for the subsequent experiments. Since no satisfactory anti-Slit2 antibody could be used to detect the endogenous Slit2 protein expression based on our preliminary study, the anti-myc antibody was used to detect the secretary Slit2 protein in the CM from HEK293 cells stably expressing Slit2-myc (Fig. 2c).

To investigate potential role of srGAP1 in CRC, we analyzed its alteration frequency in CRC using an online database tool (http://www.cbioportal.org/public-portal/index.do). Based on the data from 3 different CRC cohorts, srGAP1 locus was found to be mutated in 4% ~ 7% CRCs (Fig. 1d), whereas no deletion or amplification was observed.

### srGAP1 is a Robo1-interacting protein in CRC

**Fig. 3:**
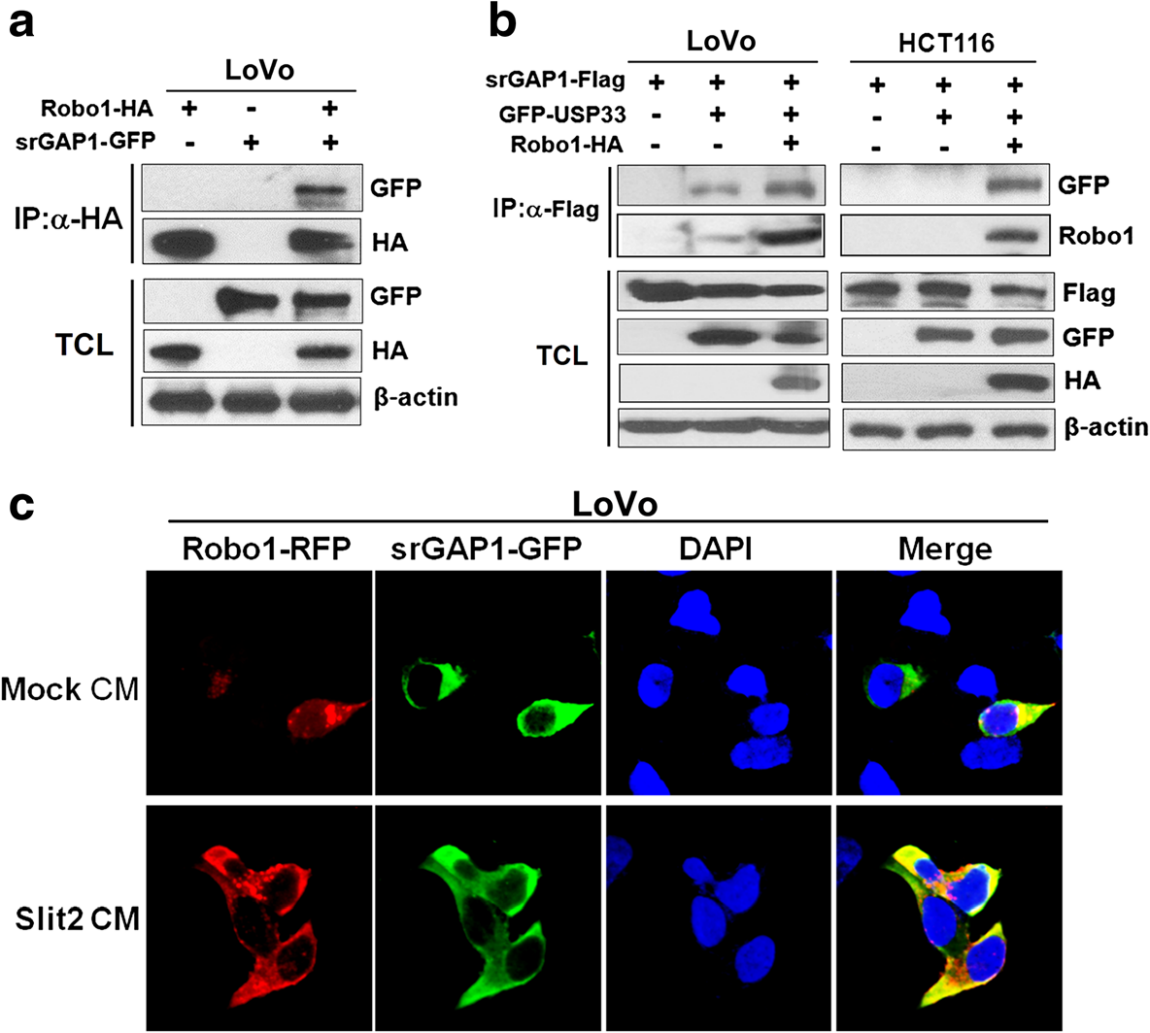
srGAP1 is a Robo1-interacting protein in CRC. **a** srGAP1 interacted with Robo1. Coimmunoprecipitation was performed to detect the interaction of Robo1-HA with srGAP1-GFP in LoVo cells transfected with Robo1-HA, srGAP1-GFP. Immunoprecipitates and total cell lysates (TCL) were immunoblotted with antibodies as indicated. **b** srGAP1 could indirectly interact with USP33 through Robo1. Coimmunoprecipitation was performed to detect the interaction of srGAP1-GFP with Robo1, GFP-USP33 in LoVo and HCT116 cells. srGAP1 could interact with USP33 in LoVo cells expressing endogenetic Robo1 but not in HCT116 cells without endogenetic Robo1 expression. **c** srGAP1 showed similar subcelluar localization as Robo1 in Slit2-treated CRC cells

Previous studies have revealed that srGAP1 could interact with Robo1 in neuron [10, 12]. To check potential interaction between Robo1 and srGAP1 in CRC cells, we performed CoIP assay. Plasmids of Robo1-HA, srGAP1-GFP or empty vector were transfected into LoVo cells. After IP using an anti-HA antibody, srGAP1 was detected in the immunoprecipitates of the cells expressing both Robo1-HA and srGAP1-GFP proteins, but not that of the control cells (Fig. 3a). To further confirm it, we performed CoIP using an anti-Flag antibody in LoVo and HCT116 cells cotranfected with Robo1-HA, srGAP1-Flag and/or USP33-GFP (also a Robo1-interacting protein). CoIP results showed that Robo1 and USP33 could be detected in the immunoprecipitates of LoVo cells expressing endogenous Robo1 but not in those of HCT116 cells without endogenous Robo1 expression (Fig. 3b). These results confirmed that Robo1 could interact with srGAP1 and USP33 in CRC cells, and srGAP1 could not interact with USP33 directly.

To check whether srGAP1 show similar subcellular localization as Robo1 in CRC cells, we performed immunofluorescence assay by cotransfecting Robo1-RFP and srGAP1-GFP plasmids into LoVo cells. As shown in Fig. 3c, most of Robo1-RFP and srGAP1-GFP were distributed in the perinuclear compartment, and only a low ratio of fluorescence (RFP or GFP) was observed at the plasma membrane in the Mock-treated cells. Whereas the fluorescence signals become stronger at the plasma membrane after Slit2 treatment, suggesting that Slit2 could initiate the redistribution of Robo1 and srGAP1 to the plasma membrane, which is consistent with our previous report [7]. Collectively, IF results show that srGAP1 and Robo1 have similar subcellular localization in the Slit2-treated CRC cells.

### Slit2-Robo1 inhibits Cdc42 activity through srGAP1

**Fig. 4:**
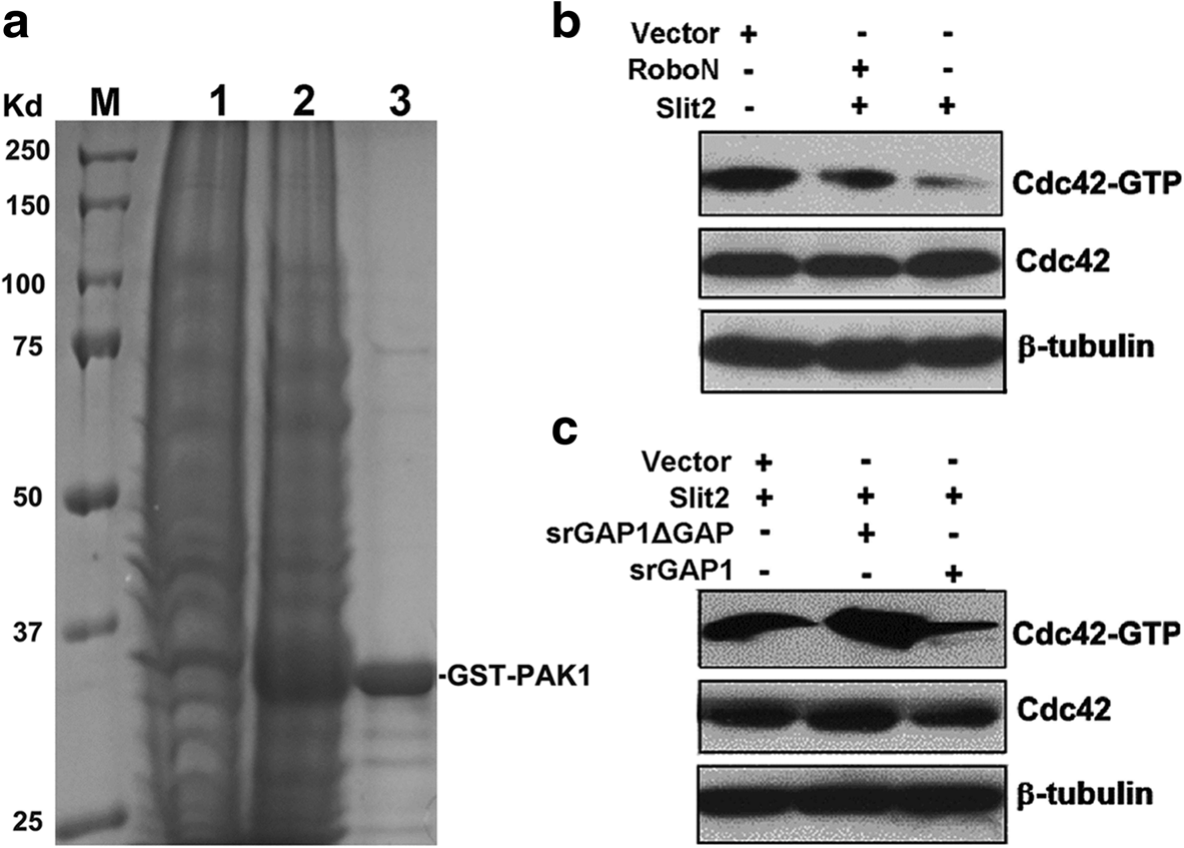
Slit2 inhibits Cdc42 activity through srGAP1. **a** Purification of GST-PAK1 protein. GST-PAK1 fusion proteins were expressed in E. coli (BL21 strain) and purified using GST-sepharose beads. M: protein marker; Total protein from control BL21 strain(1) and BL21 strain expressing GST-PAK1(2); 3: Purified GST-PAK1 protein. **b** Slit2 inhibited Cdc42 activity in a Robo-dependent manner. Ectopic expression of Slit2 inhibited Cdc42 activity (Cdc42-GTP) in LoVo cells, which could be blocked by ectopic expression of RoboN. GTP-loading of Cdc42 (GTP-Cdc42) in LoVo cell extracts was pulled down using GST-sepharose beads loaded with GST-PAK1 protein and was detected by Western blot using an anti-Cdc42 antibody. **c** Slit2 inhibited Cdc42 activity through srGAP1. Ectopic expression of srGAP1 enhanced the inhibitory effect of Slit2 on the GTPase of Cdc42, whereas blocking srGAP1 activity by srGAP1ΔGAP rescued the Cdc42-GTP levels in Slit2-overexpressed LoVo cells

Cdc42, a key member of Rho GTPases, could prompt cell migration by regulating actin cytoskeleton. Previous work revealed that Slit2 could increase srGAP1-Robo1 interaction and inactivate Cdc42, resulting in decreased cell motility in neuronal system [10]. So we checked the GTPase activity of Cdc42 in LoVo cells transfected with Slit2, RoboN, srGAP1-GFP, or srGAP1ΔGAP using GST pull-down assay (Fig. 4a). The results showed that the ectopic expression of Slit2 decrease the levels of Cdc42-GTP, which could be blocked by RoboN, suggesting that the effect is mediated by Robo (Fig. 4b). Similar results were obtained by treatment with Slit2 and RoboN CM (data not shown).

We then checked the role of srGAP1 in the regulation of Cdc42 by Slit2-Robo1 signaling. As shown in Fig. 4c, overexpression of srGAP1 could enhance the inhibitory effect of Slit2 on Cdc42 activity, whereas inhibiting of srGAP1 could block Slit2-mediated inactivation of Cdc42. Taken together, these results demonstrate that Slit2-Robo1 signaling inhibits Cdc42 activity through srGAP1.

### srGAP1 mediates the migration inhibition function of Slit2 by inactivating Cdc42 activity in CRC

**Fig. 5:**
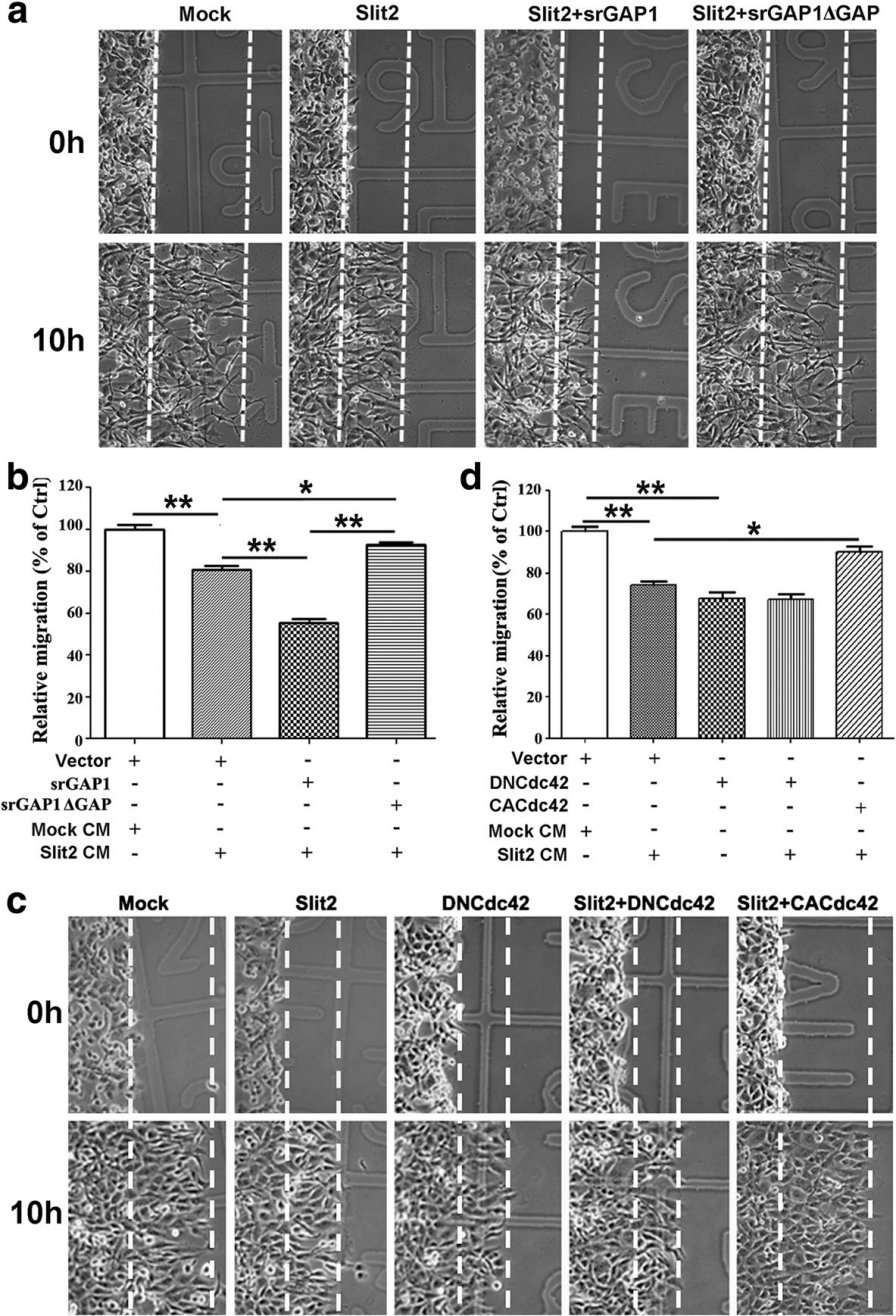
srGAP1 mediates the migration inhibitory function of Slit2 by inhibiting Cdc42 activity in CRC cells. **a-b** Slit2 inhibits CRC motility through srGAP1. Ectopic expression of srGAP1 enhanced the inhibitory effect of Slit2 on the migration of LoVo cells, whereas blocking the srGAP1 activity by srGAP1ΔGAP rescued the migration ability of Slit-treated LoVo cells. **c-d** Cdc42 is a major functional target of the Slit2-Robo1 signaling in inhibiting CRC cell migration. Blocking Cdc42 activity using DNCdc42 (dominant negative) mimicked the inhibitory effect of Slit2 on the CRC cell migration, whereas ectopic expression of CACdc42 (constitutively active) retrieved the decreased cell motility by Slit2 treatment. *P < 0.001; **P < 0.0001

Our previous work reveals that Slit2 inhibits CRC cell migration in a Robo-dependent manner [7]. To check whether the inhibitory effect of Slit2 is mediated by srGAP1 in CRC, plasmids of srGAP1 or its dominant negative mutant (srGAP1ΔGAP) were transfected into LoVo cells. Migration assay showed that ectopic srGAP1 expression significantly enhanced the migration inhibition effect of Slit2, whereas srGAP1ΔGAP blocked it (Fig. 5a-b) in LoVo cells. In addition, similar results were observed in a Transwell assay (data not shown). Collectively, these data suggest that srGAP1 is a key downstream effector of Slit2-Robo1 signaling.

Further study showed that blocking Cdc42 activity using a dominant negative form of Cdc42 (DNCdc42) could mimic the migration inhibition effect of Slit2, whereas ectopic expression of CACdc42 (constitutively active) could retrieve the decreased cell motility in Slit2-treated CRC cells, suggesting that Cdc42 is a major functional target of Slit2-Robo1-srGAP1 signaling in inhibiting CRC cell migration (Fig. 5c-d).

## Discussion

Slit-Robo signaling plays extensive role in a variety of tissue types besides theirs key role in nervous system [3, 13]. We previously revealed that Slit2 is downregulated in CRC and inhibits CRC cell migration [7]. In this study, we revealed that srGAP1 is significantly downregulated in CRC tissues, and was associated with tumor progression and survival. Further functional and mechanic analyses identified that srGAP1 mediates the migration inhibition function of Slit2 by suppressing Cdc42 activity in CRC.

Slits are important modulators of cell migration both in neuronal and non-neuronal cells. Previous studies have showed that Slit2-Robo1 pathway was frequently inactivated in human cancers, including CRC [7, 8]. Our previous data showed that Slit2 exerts a migration inhibitory role in CRC in a Robo-dependent manner, and Slit2 is downregulated in CRC due to promoter hypermethylation. Similar effect of Slit2 on cell migration of CRC also has been reported by others [9].

The underlying mechanism that Slit2 regulates tumor development and progression is very complicated [3]. Several downstream effectors, including Abl [14], Dock [15], Ena (32), ERK1/2 [16], small Rho GTPases [10], USP33 [7] and Vilse [17], have been reported to mediate the inhibitory effect of Slit2-Robo1 signaling on cell motility, proliferation, apoptosis and angiogenesis in different cell types.

A role for Rho GTPase-activating proteins in repulsion mediated by Robo has been shown in neuronal migration [10] and glioma cell invasion [11]. Small GTPases of the Rho family, including RhoA, Rac1 and Cdc42, are key regulators of actin cytoskeletal dynamics in mammals [18, 19]. These GTPases switch between an inactive status (GDP-bound) and an active status (GTP-bound), and guanine nucleotide exchange factors activate Rho GTPases while GAPs inactivate them. The binding of Slit2 modifies the cytoplasmic domains of the Robo1, and prompts the recruitment of srGAP1 to the intracellular domain of Robo1 and the inactivation of Cdc42 in neuron, suggesting srGAP1-Cdc42 a key regulatory axis of Slit2-Robo1 signaling in neuronal migration [10]. In this study, we confirmed that srGAP1 binds to Robo1 in responding to the Slit2 treatment and mediates its tumor suppressive function in CRC. Interestingly, a recent study reported srGAP1 as a candidate susceptibility gene in papillary thyroid carcinoma [20], and several mutations of srGAP1 identified in papillary thyroid carcinoma resulted in severely impaired ability of srGAP1 to inactivate CDC42. Our analysis also observed srGAP1 mutations in a fraction of CRC tumors and suggested potential roles of srGAP1 in CRC tumorigenesis, which deserves further investigations.

**Fig. 6:**
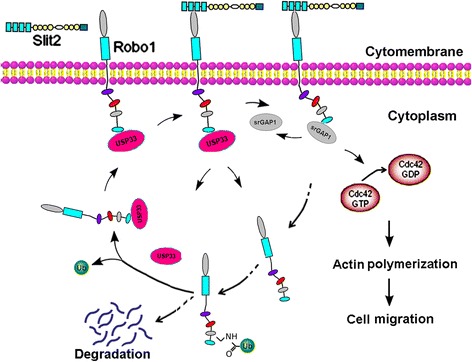
Schematic diagram of the mechanism by which srGAP1 mediating the tumor-suppressive activity of Slit2 in CRC. Slit2 inhibits CRC cell migration through the srGAP1-Cdc42 axis, and USP33 is required for the inhibitory function of Slit2-Robo1 by deubiquitinating and stabilizing Robo1 as well as taking part in the redistribution of Robo1 to the plasma membrane stimulated by Slit2

Due to the key regulatory role of Cdc42 in actin cytoskeletal dynamics, many studies have demonstrated its importance in mediating tumor aggression, and propose it a promising target of cancer therapy [21]. That CDC42 prompts cell motility has been reported in many types of human cancers [11, 19, 22–30], especially in CRC [31–34]. In this study, we revealed that Slit2 inhibited CRC cell migration via repressing Cdc42 activity. When Robo1 or srGAP1 activity was blocked, the CDC42 activity was rescued in Slit2-treated CRC cells. At the same time, blocking Cdc42 activity could mimic the inhibitory effect of Slit2 on CRC migration, whereas ectopic expression of CACdc42 could retrieve the decreased cell motility induced by Slit2. These data clearly demonstrate that the Slit2-Robo1 signaling inhibits Cdc42 activity and CRC cell motility through srGAP1, confirming the key functional role of srGAP1-Cdc42 axis in the Slit2-Robo1 signaling in CRC. In glioma and esophageal cancer, negative regulation of Slit2 on Cdc42 and cell migration also had been reported [11, 29], suggesting Cdc42 a common key target of the Slit2-Robo1 signaling in human cancers. Based on these data and our previous study [35], we propose a working model for the Slit2-Robo1 signaling transduction pathway in CRC cells (Fig. 6).

Although srGAP1 plays an important role in tumor progression, little was known about its expression and clinical significance in human cancers, including CRC. Our data showed, for the first time, that srGAP1 expression was significantly downregulated in CRC tissues. Decreased expression of srGAP1 in CRC tissues was associated with several key features of tumor aggression and progression, including lymphatic invasion, tumor differentiation and TNM stage. Furthermore, srGAP1 was identified as a potential predictor for patient survival. These data were in concordance with its role in mediating the inhibitory function of Slit2 signaling on the CRC cell motility and metastasis [7].

## Conclusion

In summary, our data revealed that the downregulation of srGAP1 in CRC tissues is associated with tumor progression and poor prognosis, and srGAP1-Cdc42 regulatory axis is a key downstream target of Slit2-Robo1 pathway in CRC. Slit2-Robo1-srGAP1- Cdc42 pathway appears to be a promising treatment target for CRC, and deserves further investigations.

## Abbreviations

CI: Confidence interval
CM: Conditioned medium
CRC: Colorectal cancer
FBS: Fetal bovine serum
HR: Hazard ratio
IF: Immunofluorescence
IHC: Immunohistochemistry
IP: Immunoprecipitation
NCT: Adjacent noncancerous tissue
OS: Overall survival
srGAP1: The Slit-Robo Rho GTPase activating protein 1
